# Neural changes following a body-oriented resilience therapy with elements of kickboxing for individuals with a psychotic disorder: a randomized controlled trial

**DOI:** 10.1007/s00406-020-01097-z

**Published:** 2020-01-24

**Authors:** Elisabeth C. D. van der Stouwe, Gerdina H. M. Pijnenborg, Esther M. Opmeer, Bertine de Vries, Jan-Bernard C. Marsman, André Aleman, Jooske T. van Busschbach

**Affiliations:** 1 University of Groningen, University Medical Center Groningen, University Center of Psychiatry, Rob Giel Onderzoekcentrum, Hanzeplein 1, 9713 GZ Groningen, The Netherlands; 2 Department of Neuroscience, Cognitive Neuroscience Center, University of Groningen, University Medical Center Groningen, Antonius Deusinglaan 2, 9713 AW Groningen, The Netherlands; 3 Department of Clinical Psychology, University of Groningen, Grote Kruisstraat 2/1, 9712 TS Groningen, The Netherlands; 4 Department of Psychotic Disorders, GGZ-Drenthe, Dennenweg 9, 9404 LA Assen, The Netherlands; 5 Department of Health and Social Work, Windesheim University of Applied Sciences, Campus 2-6, 8017 CA Zwolle, The Netherlands; 6 Department of Movement and Education, Windesheim University of Applied Sciences, Campus 2-6, 8017 CA Zwolle, The Netherlands

**Keywords:** Body oriented, Face processing, fMRI, Kickboxing, Psychosis, Social cognition, Victimization

## Abstract

Individuals with a psychotic disorder are at an increased risk of becoming the victim of a crime. A body-oriented resilience therapy (BEATVIC) aimed at preventing victimization by addressing putatively underlying factors was developed. One of these factors is social cognition, particularly facial affect processing. The current study investigated neural effects of BEATVIC on facial affect processing using two face processing tasks. Participants were randomized to either BEATVIC or a ‘Befriending’ control group. Twenty-seven patients completed an Emotional Faces task and the Wall of Faces task during fMRI, pre- and post-intervention. General linear model analyses yielded no differences between groups over time. Independent component analyses revealed increased activation of the salience network to angry and fearful faces in BEATVIC compared to Befriending. Increased activation of the salience network may suggest an increased alertness for potentially dangerous faces.

**Electronic supplementary material:**

The online version of this article (10.1007/s00406-020-01097-z) contains supplementary material, which is available to authorized users.

## Introduction

Individuals diagnosed with a psychotic spectrum disorder are more susceptible to become the victim of a crime than people from the general population [1, 2]. Victimization can have a considerable impact on peoples’ lives, leading to for example substance abuse, depression [3], more severe symptomatology and poorer illness outcome [4]. To decrease the risk of victimization in people with a psychotic disorder, a body-oriented resilience therapy was developed, henceforward referred to as BEATVIC [5]. BEATVIC aims to prevent victimization by addressing associated factors which are modifiable and feasible to improve by means of an intervention.

One of these risk factors is impaired social cognitive deficits [6], such as problems in processing facial expressions that often accompany psychotic disorders [6, 7]. Individuals with a psychotic disorder often show a deficiency in recognizing facial expressions, body language, mentalization and prosody which could prevent accurate judgement of threatening social situations which may ultimately result in victimization. These deficits in social cognitive functioning have been acknowledged as an important treatment target to help patients recognize and manage potentially threatening situations and to adopt self-protective behaviors to reduce their risk of victimization [8]. Especially processing of facial expressions might be relevant in the context of victimization, because adequate processing of faces enables recognizing the intentions of potential perpetrators. In BEATVIC, by practising kickboxing techniques with a partner, and by observing others in a group and discussing afterward, participants learn to identify (threatening) non-verbal communication, such as negative facial expressions, body postures and gestures. In addition, participants are encouraged to reflect on their own behavior gaining insight into how they appear to others, and accordingly they can experiment with new behavior in a safe therapeutic environment. For a comprehensive explanation of BEATVIC, see [5, 9].

The neural correlates of effects of therapeutic interventions such as BEATVIC can have important implications for our understanding of the mechanisms of therapeutic change [10, 11]. While the current study was the first to investigate this specific therapy, several previous studies looked at neural effects of social cognition training (SCT), an intervention which also targets emotional face processing. A review of neural changes following SCT in people with psychotic disorders revealed normalizing effects in key areas involved in emotional facial processing: early visual perceptual regions, prefrontal gyri, and the amygdala and insula [12]. While early visual processing areas are known to show hypo-activation and reduced volume in psychosis, studies found increased activation in the posterior parietal and occipital cortex in response to face processing [13] and reduced gray matter volume loss in the fusiform gyrus [14] after SCT in schizophrenia. Similarly, whereas psychotic disorders were associated with reduced activation in the frontal regions during face processing [15], studies have revealed increased activity in the inferior, medial and/or superior frontal gyrus over time following SCT [16, 17]. Finally, the activity of the insula and amygdala, which is decreased in psychosis during facial expression recognition, was increased following SCT [13, 14, 18]. In conclusion, previous studies have revealed meaningful neural effects of SCT in patients with a psychotic disorder.

The aim of the present study was to examine neural changes in areas associated with face processing following a body-oriented resilience therapy targeting factors associated with victimization, such as social cognition, in individuals with a psychotic disorder. To achieve this, we studied the effect of BEATVIC on functional activation of the brain during processing of faces denoting direct as well as indirect threat [19, 20]. Angry expressions signal a direct and immediate threat from a potential perpetrator, while fearful expressions indicate a possible presence of a significant source of threat in the environment, as witnessed by others [20]. Especially processing of threatening facial expressions might be relevant in the context of victimization, because adequate processing of angry faces enables recognizing the intentions of potential perpetrators, whereas adequate processing of fearful faces enables detecting this indirectly. A first study comparing a group of recently victimized participants with a psychotic disorder and patients who had not been victimized revealed more deactivation of the sensorimotor network during processing of angry faces (van der Stouwe et al., submitted).

Earlier studies on SCT in psychosis reported normalizing effects on early visual processing areas, frontal areas, and facial expression recognition areas, the insula and amygdala. Therefore it was hypothesized that BEATVIC would lead to increased activation in these areas as well. Since our previous study on baseline data of this study revealed stronger deactivation of the sensorimotor network during processing of angry faces in a victimized group of patients, we additionally explored the effect of BEATVIC on the sensorimotor network.

## Methods

### Participants

A total number of 41 participants were recruited from five mental health institutions in the Netherlands as part of the larger ‘Beat victimization’ study (BEATVIC; [5]). Inclusion criteria were: age ≥ 18 years and a diagnosis in the psychotic spectrum according to DSM-IV-TR. Exclusion criteria were: severe psychotic symptoms (PANSS mean positive symptoms > 5), substance dependence (not substance abuse), co-morbid neurological disorder, co-morbid personality disorder, estimated IQ < 70, pregnancy and MRI incompatibility. To verify whether participants met the inclusion criteria, trained interviewers completed a mini-SCAN interview [21], a PANSS interview [22] and an MRI safety checklist. The study was approved by the local ethical committee (University Medical Center of Groningen, The Netherlands; METc protocol number: NL52202.042.15) and was performed in line with the Declaration of Helsinki. Written informed consent was obtained from all participants. All patients from the trial were randomly assigned to one of the two treatment arms, BEATVIC or Befriending (Current Controlled Trials: ISRCTN21423535), by an independent researcher. Randomization was carried out for each site separately to guarantee a comparable number of participants in both groups. Randomization was stratified by gender and participation in the fMRI substudy, to ensure that the treatment groups were balanced in respect of gender distribution and to make sure the number of participants in both treatment arms was balanced in the fMRI substudy.

### Intervention

#### BEATVIC

BEATVIC consists of 20 weekly group sessions of 75 min led by a therapist trained in body and movement-oriented interventions (in the European literature called a psychomotor therapist; see www.psychomot.org/) and an expert by experience. Each session includes a warming up, technical kickboxing exercises and thematic exercises, a cooling down and a discussion of the addressed factors to make a transfer to daily life. The 20 sessions are divided into five modules.

In the first module, kickboxing techniques are introduced with a special emphasis on self-stigma and setting and respecting boundaries. The second module, ‘Recognizing dangerous behavior’, aims to increase social cognition by identifying (threatening) non-verbal communication, such as body postures, gestures and facial expressions. Whereas this module focuses on interpretation of the behavior of others, the third module, ‘How others see me’, emphasizes patients’ own behavior. The aim is to gain insight into factors which affect one’s own behavior (e.g., emotions, characteristics of someone else) and insight into automatic natural reactions to dangerous situations (e.g., fight, flight or fright). Special emphasis is on the role of body posture, balance, voice and breath to feel and appear stronger. The aim of the fourth module ‘Coping with aggression’ is to learn to detect and regulate one’s own aggression, but also to learn to deal with the aggressive behavior of others. Exercises focus on bodily signals of anger and tension and ways to reduce tension. Observational exercises are used to detect signals of tension in others. Throughout BEATVIC, couple exercises and observational exercises are implemented to practise reading others’ behavior. In the last module, exercises that were particularly useful for the specific group are repeated.

#### Control group

To control for structural weekly social contact in a group setting, the control group was offered 20 weekly ‘Befriending’ group sessions of 75 min. The aim of these sessions is to provide a welcoming atmosphere in which participants can socially interact in an informal setting. Befriending consists of five modules ‘Introduction’, ‘Media’, ‘Hobbies’, ‘Lifestyle’ and ‘Repetition and follow-up’. Throughout these modules, groups for example play board games, discuss the news, watch a documentary, cook a meal, discuss their hobbies or make Christmas cards. Trainers make sure only neutral topics, such as music, books or sports, are discussed. Befriending has been used as a control treatment in several studies investigating cognitive behavior therapy in the treatment for psychosis [23].

### Measures

Before and after the intervention period, participants underwent functional and structural MRI scanning.

#### Emotional Faces (EF) task

With the Emotional Faces (EF) task, brain response to threatening emotional faces was investigated. Participants completed a gender discrimination task including 16 blocks of individual angry, neutral, happy and fearful faces [24]. Each block contained six trials, including three to five face trials from one emotion condition and one to three null trials consisting of a fixation cross. Faces and null trials were randomly mixed within blocks. Each face trial consisted of a stimulus presented for 600 ms and an interstimulus interval of 200 ms during which a fixation cross was displayed. Participants were instructed to respond (indicate the gender) by means of a button box as fast as possible.

#### Wall of Faces (WoF) task

The WoF task [25] enables investigating brain response to a group of predominantly angry faces containing trials, each presenting an array of 32 emotional faces (i.e., angry or happy). Participants were asked to indicate the predominant emotion (emotion blocks, experimental condition) or the predominant gender (gender blocks, control condition) of the array of faces. The ratio of angry to happy faces and male to female faces could be equal (ambiguous, 16:16) or unequal (unambiguous, 26:6). In each trial, the 32 faces were presented for 3 s, followed by a 1.5 s response time. During face presentation and response time, the options “Angry–Happy” or “Female–Male” were displayed on the screen. Blocks of eight trials (48 s) started with an instruction (“emotion” or “gender”) and were interleaved with a fixation cross (24 s). Emotion and gender blocks were alternated.

### Magnetic resonance imaging acquisition

Neuroimaging data were acquired on a 3 T Philips Intera MR-scanner (Best, The Netherlands), equipped with a 32-channel SENSE head coil. During the task, whole-brain functional images were acquired using a T2*-weighted echo-planar sequence (39 descending axial slices; slice thickness = 3 mm; slice gap = 0 mm; TR = 2000 ms; TE = 30 ms; FOV 192 × 192 × 117 mm; voxel size = 3 mm isotropic; flip angle = 90 degrees; 275 volumes). All scans were oriented approximately 10°–20° to the AC–PC transverse plane to prevent artifacts due to nasal cavities. In addition, a high-resolution anatomical T1 image was recorded (170 slices; slice thickness = 1 mm; TR = 9 ms; TE = 3.5 ms; FOV 256 × 232 × 170 mm; voxel size 1 mm isotropic).

### Statistical analyses

#### Demographic characteristics

Demographic and clinical differences between the treatment and control group were tested using a Pearson Chi-squared test for categorical variables or Fisher’s exact tests in case expected cell counts < 5. Continuous variables were tested with independent *T* tests and Mann–Whitney *U* tests. Because depression and paranoia may influence processing of facial expressions [26, 27], we also explored group differences on the individual PANSS depression item and paranoia item.

#### Behavioral data

For the EF and the WoF task, reaction times (RT) were analyzed by a Group (BEATVIC, befriending)  ×  Time (pre-assessment, post-assessment)  ×  Condition (angry, fearful or unambiguous_moreangry, unambiguous_emotion, ambiguous_emotion) RM ANOVA with Group defined as a between-subject factor and Time and Condition as the within-subject factors. Accuracies (Accs) were analyzed by means of a similar RM ANOVA, but for the WoF task only the responses to the unambiguous trials were used, as there were no correct responses in the ambiguous trials.

#### Preprocessing

Neuroimaging data were preprocessed and analyzed using Statistical Parametric Mapping 12 version 6470 (Welcome Department of Cognitive Neurology, UCL) in Matlab version 7.8.0 (Mathworks, Natick USA). First, T1 and T2* images were reoriented manually to the AC–PC plane. Functional images were then realigned and co-registered to the anatomical T1 image. Next, the data were normalized to Montreal Neurological Institute (MNI) space. Finally, images were smoothed using an 8 mm full width half maximum Gaussian kernel.

#### Voxel-based morphometry analyses

Because we were primarily interested in functional neural changes related to face processing, we chose to control for potential effects of the amount of exercise in BEATVIC, if any such effects were apparent. A review by our group [21] has shown that previous research on the neural correlates of physical activity interventions in psychosis mostly focused on the hippocampus, often revealing an increase in hippocampal volume [22, 23] or a dose-dependent prevention of hippocampal volume decline over time [24]. However, it was concluded that an average weekly exercise frequency of at least two times a week may be the minimum to detect neural changes of physical activity interventions in psychosis [25]. Therefore no volume changes, measured by means of voxel-based morphometry (VBM), were expected but in case of changes these were controlled for.

T1 images were segmented into gray matter, white matter, cerebrospinal fluid, bone, soft tissue, air/background. The Diffeomorphic Anatomical Registration Through Exponentiated Lie algebra (DARTEL) approach was used for optimal registration of individual segments to a group mean template. The DARTEL-normalized modulated and unmodulated gray matter segments were further normalized to the Montreal Neurological Institute (MNI) space. The left and right hippocampus, defined by the Automated Anatomic Labelling system implemented in the Wake Forest University PickAtlas (https://fmri.wfubmc.edu/software/PickAtlas), was used as regions of interest (ROIs). Gray matter volume, white matter volume, intracranial volume, bilateral hippocampus volume, left hippocampus volume and right hippocampus volume (in ml) were computed with in-house scripts and entered in SPSS. An RM ANOVA was used to investigate Group (BEATVIC, Befriending)  ×  Time (pre-assessment, post-assessment) effects.

#### GLM activation analyses

Pre- and post-neuroimaging data were entered together in first-level models. For the EF task, four task regressors (angry, neutral, happy, fearful), defined as onset times per trial, were convolved with the canonical hemodynamic response function. For the WoF, six task conditions (ambiguous emotion, ambiguous gender, unambiguous more happy faces, unambiguous more angry faces, unambiguous more female faces, unambiguous more male faces) and an instruction condition (notifying task and resting blocks) were modeled. To correct for motion, six motion parameters and their first derivatives were added to all models. In addition, frame-wise displacement (FD) was calculated and included as a regressor. Motion was deemed excessive when FD > 0.9 for a certain volume (Siegel et al. 2014). Because we were interested in a potential change in threat response over time, we created the EF contrasts: t1(angry>baseline)>t2(angry>baseline) and t1(fear>baseline)>t2(fear>baseline). For the WoF, the following contrasts were computed: t1(unambiguous_more_angry_faces>unambiguous_more_happy_faces)>t2(more_angry>more_happy), t1(ambiguous_emotion>unambiguous_emotion)>t2(amb_emo>unamb_emo), t1(ambiguous_trials>unambiguous_trials)>t2(amb>unamb).

Single-subject contrast images of pre-treatment data only were used to perform one-sample *t* tests at second level to examine the main task effects. Two-sample *t* tests were performed to compare the differences over time for the treatment and the control group. Medication use was entered as covariate of no interest in all analyses by means of a dummy variable (yes/no antipsychotic medication). All tests were performed at an initial threshold of *p* < 0.001 with FWE cluster correction at *p* < 0.05.

#### Independent component analysis

Independent component analysis (ICA) was performed with the Group ICA of fMRI Toolbox (GIFT; version 3.0a, MIALAB Software) [28], which was implemented in Matlab version 7.8.0. Both pre- and post-functional time series were entered into ICA. The number of independent components was estimated using maximum description length (MDL) and Akaike’s criteria, which resulted in 32 components for both the EF and WoF task. For all participants, images were decomposed into 32 spatially independent components using the Infomax algorithm. Single-subject time courses and spatial maps were back-reconstructed by means of spatial–temporal regression. Subsequently, a group ICA was performed and its stability was assessed by performing an ICASSO on 20 iterations [29].

To select components, for both tasks the correlation between the time course of the independent components and the conditions of the task was determined. The design matrices derived from the GLM analyses were entered in the temporal sorting function (multiple regression) in GIFT. To make sure we selected components including our brain areas of interest, three anatomical masks containing, respectively, early visual processing areas (fusiform gyrus, inferior/middle/superior occipital gyrus), frontal areas (inferior/middle/superior frontal gyrus) and facial expression recognition areas (the insula and amygdala) were created with WFU-pickatlas (https://www.nitrc.org/projects/wfu_pickatlas). We performed a spatial sorting of all components based on each of these masks (multiple regression) in GIFT. Overall, for each ROI (visual, frontal, insula and amygdala), we selected the component with the highest correlation with the task. In addition, we selected the sensorimotor network. Components were identified based on previous resting state studies [30, 31].

Following temporal sorting, the resulting beta weights represented the amount of task-related activation or deactivation per independent component per condition, per assessment (pre- or post-assessment) for every subject. These beta weights were entered into SPSS. Change scores (post-intervention − pre-intervention) were calculated and used to determine group differences in network activation or deactivation by using the Mann–Whitney *U* test (*α* = 0.05).

## Results

### Sample characteristics

Of the 41 participants that underwent pre-treatment fMRI-scanning, 31 participants also completed the post-treatment fMRI session. A CONSORT flow diagram indicating the numbers and reasons of therapy dropout and treatment dropout can be found in Supplementary material Fig. S1. The data of two participants were excluded due to excessive head movement (> 3 mm) and the data of two participants were excluded due to technical problems. The demographic and clinical characteristics of the remaining 27 participants are depicted in Table 1. The treatment and control groups did not differ in socio-demographic characteristics or illness-related characteristics.

**Table 1:** Demographic and clinical characteristics at pre-assessment

	BEATVIC	Befriending	Test statistic
*N*	14	13	
Age, mean (SD)	32.4 (10.0)	36.4 (11.5)	*t*(26) = 0.78, *p* = 0.37
Gender, *N*(%) male	9 (64.3)	10 (76.9)	*X*^*2*^(1) = 0.52, *p* = 0.47
*Occupational status, N(%)*			*p* = 1.00
Job	4 (28.6)	3 (23.1)	
Voluntary work	2 (14.3)	2 (15.4)	
Unemployed	8 (57.1)	8 (61.5)	
*Living situation, N(%)*			
Alone	7 (50)	8 (61.5)	*p* = 0.92
Partner	1 (7.2)	1 (7.7)	
Family/parents	4 (28.6)	2 (15.4)	
Supported housing	2 (14.3)	2 (15.4)	
Age of onset, mean (SD)	19.1 (6.2)	21.5 (8.3)	*t*(26) = − 0.85*, p* = 0.26
Number of psychotic episodes, mean (SD)	3.8 (3.9)	4.3 (3.8)	*t*(26) = − 0.35*, p* = .61
Number of admissions, mean (SD)	1.5 (1.4)	1.5 (1.5)	*t*(26) = − 0.07, *p* = 0.86
*PANSS score, mean (SD)*			
Total	48.9 (7.9)	50.0 (9.2)	*u* = 81.5, *p* = 0*.*64
Positive	12.6 (3.7)	12.7 (3.5)	*u* = 89, *p* = 0.92
Negative	10.6 (2.6)	11.4 (3.0)	*u* = 80.5, *p* = 0.61
General	25.7 (4.3)	25.9 (5.5)	*u* = 91, *p* = 1.0
BNSS total score	13.5 (8.1)	14.8 (7.3)	*u* = 80, *p* = 0.59
PANSS depression item	2.5 (1.6)	2.9 (1.4)	*u* = 76, *p* = 0.45
PANSS paranoia item	2.8 (2.8)	2.2 (1.1)	*u* = 87.5, *p* = 0.86
Antipsychotic medication, *N*(%)			*p* = 0.60^a^
Risperidone	2 (14.3)	3 (23.1)	
Olanzapine	2 (14.3)	2 (15.4)	
Clozapine	3 (21.4)	3 (23.1)	
Aripiprazole	3 (21.4)	2 (15.4)	
Quetiapine	2 (14.3)	1 (7.7)	
Haloperidol	2 (14.3)	1 (7.7)	
Paliperidone		2 (15.4)	
Penfluridol		1 (7.7)	
None	3 (21.4)	1 (7.7)	
*Antidepressant medication, N(%)*			*p* = 0.13^a^
Citalopram	1 (7.2)	4 (30.8)	
Venlafaxine	2 (14.3)	1 (7.7)	
Amitriptyline	1 (7.2)		
Nortriptyline		1 (7.7)	
Lithium	2 (14.3)	1 (7.7)	
*Clomipramine*			
Mirtazapine		2 (15.4)	
None	10 (71.4)	5 (38.5)	

^a^Fisher’s exact test to check for differences in medication use (yes/no) between both groups

### Behavioral results

For the Emotional Faces (EF) task, groups did not differ in RT and Acc of the angry and fearful face conditions pre-treatment. An RM ANOVA revealed no significant differences between groups over time. Similarly, with regard to the WoF task, groups did not differ in RT and Acc pre-treatment and there were no significant Group by Time interactions (see Table 2).

**Table 2:** Mean reaction times (RT) and accuracies (Accs) at pre- and post-assessment

	BEATVIC		Befriending		*F, p*
	Pre (mean. sd)	Post (mean. sd)	Pre (mean. sd)	Post (mean. sd)	
*EF*					
RT_angry	559.2 (53.8)	546.6 (50.3)	609.1 (84.4)	589.74 (65.8)	*F*(1,26) = 0.29, *p* = 0.60
RT_fearful	563.8 (64.4)	563.46 (49.0)	606.9 (79.8)	596.21 (71.5)	*F*(1,26) = 0.36, *p* = 0.56
Acc_angry	46.5 (9.6)	53.5 (6.0)	41.6 (19.2)	48.3 (16.5)	*F*(1,26) = 0.03, *p* = 0.86
Acc_fearful	47.5 (12.3)	52.9 (6.5)	43.7 (20.4)	48.7 (17.0)	*F*(1,26) = 0.10, *p* = 0.76
*WoF*					
RT_unamb_moreangry	2264.9 (650.1)	2112.3 (706.5)	2538.1 (814.4)	2619.0 (879.0)	*F*(1,24) = 0.75, *p* = 0.40
RT_unamb_emotion	2098.7 (517.1)	2088.5 (703.4)	2476.3 (832.0)	2602.10 (761.9)	*F*(1,24) = 0.41, *p* = 0.53
RT_amb_emotion	2366.3 (525.5)	2282.2 (669.2)	2691.4 (773.8)	2705.3 (818.8)	*F*(1,24) = 0.23, *p* = 0.63
Acc_unamb_moreangry	6.4 (1.2)	7.0 (0.8)	6.6 (1.2)	5.8 (2.6)	*F*(1,24) = 2.26, *p* = 0.15
Acc_unamb_emotion	13.6 (2.0)	14.3 (1.4)	13.1 (1.9)	11.0 (5.3)	*F*(1,24) = 3.23, *p* = 0.09

### VBM analysis

Brain volumes for BEATVIC and Befriending at pre- and post-assessment are displayed in Table 3. At pre-assessment, groups did not differ in brain volume. No significant Group by Time interactions were found. Hence, brain volume was not included as a covariate in further analyses.

**Table 3:** Brain volumes at pre- and post-assessment

	BEATVIC		Befriending		*F, p*
	Pre	Post	Pre	Post	
Gray matter	729.38 (89.51)	722.22 (96.86)	732.63 (120.02)	726.28 (123.78)	*F*(1,25) = 0.02, *p* = 0.89
White matter	466.83 (42.38)	468.67 (39.12)	474.04 (57.74)	478.12 (57.5)	*F*(1,25) = 0.57, *p* = 0.46
Intracranial volume	1403.29 (96.65)	1396.51 (135.51)	1483.28 (152.96)	1474.92 (169.68)	*F*(1,25) = 0.00, *p* = 095
Left hippocampus	4.68 (0.31)	4.65 (0.28)	4.60 (0.42)	4.56 (0.43)	*F*(1,25) = 0.05, *p* = 0.83
Right hippocampus	4.06 (0.37)	4.01 (0.29)	3.97 (0.37)	3.92 (0.38)	*F*(1,25) = 0.04, *p* = 0.84

^a^All volume measurements are expressed in mean and SD milliliters (ml)

### GLM activation analyses

#### Emotional Faces (EF) task

##### Task effects

The task activated occipital, frontal areas, insula and amygdala as was shown by contrasting, respectively, angry faces and fearful faces against baseline. Both contrasts revealed a similar pattern of brain regions (for overview see Supplementary material Table SII/Fig. S3).

##### Group differences

There were no significant time differences between groups: the BEATVIC group did not differ from the Befriending group on comparing the pre-treatment and post-treatment brain response to angry faces, respectively, while fearful faces contrasted with baseline.

#### Wall of Faces (WoF) task

##### Task effects

The WoF did not reveal differences in activation between task conditions when investigating all pretreatment scans with a one-sample *t* test. This may be due to the fact that the different conditions are very similar in terms of visual input (array of faces on a screen) and cognitive processes recruited during the task (cf. [32]).

##### Group differences

There were no significant group differences in time effects: groups did not differ in change in brain response over time.

### Independent component analysis

#### EF

**Fig. 1:**
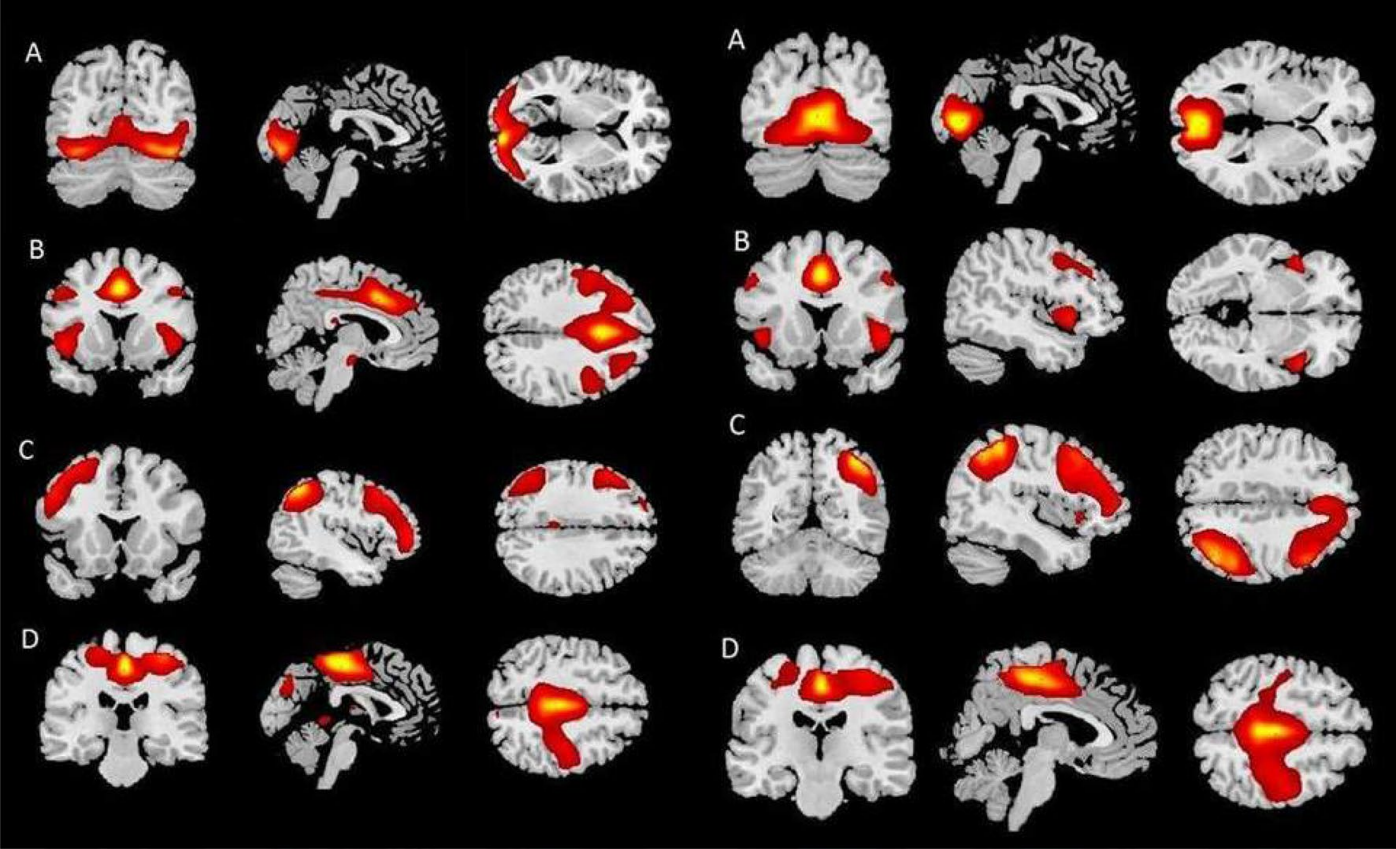
The spatial maps of selected components for the EF task (left) and WoF task (right). Left: **a** visual network, **b** salience network, **c** left frontoparietal network, **d** sensorimotor network. Right: **a** medial visual network, **b** salience network, **c** right frontoparietal network, **d** sensorimotor network

In total, 32 independent task-related network components were estimated. The component including visual regions with the highest correlation with the task (*r* = 0.41) was the visual network. The component including facial expression recognition areas with the highest correlation with the task (*r* = 0.07) comprised the salience network. The component including frontal areas with the highest correlation with the task (*r* = 0.07) was the left frontoparietal network. In addition, the sensorimotor network (*r* = 0.07) was selected. All selected components are depicted in Fig. 1.

#### WoF

In total, 32 independent task-related network components were estimated for the WoF task as well. The component including visual regions that showed the highest correlation with the task (*r* = 0.62) was the medial visual network. The component including emotional face processing areas with the highest correlation with the task (*r* = 0.21) consisted of the salience network. The component including frontal regions with the highest correlation with the task comprised the right frontoparietal network (*r* = 0.21). Finally, the sensorimotor network (*r* = 0.14) was selected. The selected components are depicted in Fig. 1 (for a detailed description of selected networks see Supplementary material S.4).

### Independent component analysis: task correlations

#### EF

The component consisting of the salience network showed significant differences in task-related network activation between groups over time during processing of both fearful (*U* = 44.00, *z* = − 2.28, *p* = 0.02) and angry faces (*U* = 33.00, *z* = − 2.82, *p* = 0.005). For fearful faces, increased activation over time was found in the BEATVIC group and decreased activation over time was found in the Befriending group. For angry faces, decreased activation over time in this network was found in the Befriending group.

The visual component showed a trend difference between groups over time during processing of angry faces (*U* = 51.00, *z* = − 1.94, *p* = 0.05). Similarly, increased activation over time during the processing of angry faces was found in the BEATVIC group, while the Befriending group showed decreased activation over time. There was also a trend Time × Group interaction effect for the sensorimotor component during fearful faces (*U* = 55, *z* = − 1.75, *p* = 0.08). This effect was due to decreased deactivation over time in BEATVIC and increased deactivation over time in Befriending. No Time × Group interactions were found for the component consisting of the left frontoparietal network.

#### WoF

The medial visual network showed a trend Time × Group interaction during processing of a stimulus comprising a wall of more angry faces than happy faces (*p* = 0.07), because Befriending showed decreased activation over time. The other selected components did not show differences between groups across time. Means and standard deviations of beta weights for each component for both groups and both assessments are depicted in Table 4.

**Table 4:** Mean beta weights per component for participants in the BEATVIC (*n* = 14) and Befriending (*n* = 13) groups. pre- and post-treatment

	BEATVIC		Befriending	
	Pre	Post	Pre	Post
EF				
Visual network				
Angry faces*	3.77 (0.70)	4.03 (1.23)	4.19 (0.65)	3.91 (1.03)
Fearful faces	3.81 (0.80)	4.00 (0.99)	4.14 (0.76)	4.06 (1.03)
Salience network				
Angry faces*	1.56 (0.82)	1.58 (0.92)	1.69 (0.53)	0.63 (0.88)
Fearful faces*	1.28 (0.92)	1.48 (0.93)	1.48 (0.71)	0.62 (0.93)
Left frontoparietal network				
Angry faces	− 0.91 (0.89)	− 1.04 (0.95)	− 0.42 (1.29)	− 0.78 (0.82)
Fearful faces	− 136 (0.96)	− 1.02 (1.06)	− 0.75 (1.16)	− 0.85 (1.01)
Sensorimotor network				
Angry faces	− 0.71 (0.84)	− 0.80 (1.49)	− 0.49 (1.31)	− 1.07 (1.09)
Fearful faces*	− 1.10 (0.90)	− 0.64 (1.25)	− 0.60 (1.30)	− 1.08 (1.19)
WoF				
Medial visual network				
More angry faces *	1.49 (1.25)	2.08 (0.31)	2.18 (0.20)	2.19 (0.20)
Ambiguous emotion trials	1.65 (1.22)	2.02 (0.26)	2.11 (0.28)	2.13 (0.31)
Salience network				
More angry faces	0.67 (0.80)	0.87 (0.39)	1.12 (0.49)	1.28 (0.39)
Ambiguous emotion trials	0.99 (0.64)	1.10 (0.35)	1.15 (0.52)	1.35 (0.26)
Right frontoparietal network				
More angry faces	0.66 (0.76)	0.72 (0.53)	1.11 (0.58)	0.98 (0.65)
Ambiguous emotion trials	0.81 (0.69)	0.84 (0.48)	1.14 (0.57)	1.22 (0.57)
Sensorimotor network				
More angry faces	− 0.61 (0.85)	− 0.49 (0.49)	− 0.58 (0.56)	− 0.92 (0.61)
Ambiguous emotion trials	− 0.78 (0.55)	− 0.58 (0.45)	− 0.82 (0.62)	− 0.98 (0.43)

^*^(Trend) significant difference between groups across time

## Discussion

The aim of this study was to examine neural changes following BEATVIC, a body-oriented resilience therapy with kickboxing exercises. After the intervention period, the BEATVIC group showed increased activation of the salience network compared to the Befriending group during processing of fearful and angry faces. No differences were found between the BEATVIC group and the Befriending group over time in terms of regional brain activation as analyzed with conventional GLM analysis.

In line with our hypothesis, using ICA investigation of networks, we found increased activation in a component that included facial expression processing areas following BEATVIC compared to Befriending. This finding resembles results of previous studies that have reported increased activation in the insula [13, 14, 18] during Emotional Faces tasks following SCT. Several meta-analyses have found reduced activation in the insula in schizophrenia and psychosis [15, 33]. This might indicate that BEATVIC normalizes activation in the salience network; however, to confirm this, a future study including a healthy control group is needed. As the salience network is involved in detecting and filtering salient stimuli [34], increased activation of this network may suggest that BEATVIC results in better detection of salient information from the environment. Patients might have become more alert to threatening or potential dangerous faces. However, such interpretations would need to be corroborated with behavioral evidence and thus replication in larger groups is needed.

Contrary to our hypothesis, the frontal component did not reveal differential activation between groups over time. Whereas BEATVIC also consists of reflection on one’s feelings and behavior and exercises in which participants have to dose and control their own strength, the intervention is primarily non-verbal and experience-based, including many exercises that evoke behavioral reflexes which involves processes that may not recruit frontal brain regions. Indeed, previous SCT studies reporting effects on frontal regions often included cognitive training [17, 35] which, rather than basic perceptual face processing related processes, might have been responsible for frontal activation. Also contrary to our hypothesis, we found no significant effect of BEATVIC on the visual network. However, although no formal significance was reached, the (medial) visual network did show a trend toward significance (*p* = 0.05). More research with larger sample sizes is warranted to allow for more definite conclusions. As the (medial) visual network is implicated in processing of visual stimuli, increased activation of this network might indicate that BEATVIC may lead to processing faces more adequately. Indeed, the descriptives of the behavioral data of the WoF show more accurate responses over time for BEATVIC and less accurate responses following Befriending.

In addition to the visual network, frontal network and salience network, we explored whether there was an effect on the sensorimotor network between groups over time. Although not significant, a trend (*p* < 0.08) for decreased deactivation in the sensorimotor network in response to fearful faces in BEATVIC compared to Befriending warrants future research. In earlier analyses of the baseline data of these patients, we found more deactivation of the sensorimotor network in those patients who had a history of recent victimization [36]. It may be clinically relevant if BEATVIC could potentially invert this deactivation. The sensorimotor network is implicated in preparation and execution of actions. Decreased activation in sensorimotor regions and decreased connectivity within the sensorimotor network have been associated with the common symptom ‘freezing of gait’ in patients with Parkinson’s disease which refers to a brief, involuntary abortion of movement [37, 38]. Deactivation of the sensorimotor network in victimized participants may resemble to some extent the freeze response reported in traumatized individuals in response to threat [39, 40]. In case future research would provide further evidence for deactivation of the sensorimotor network, it could be speculated that BEATVIC might lead to the tendency to freeze less and undertake action instead in response to indirect threat, which might be explained by the physical activation (and exercises that address reflexes such as fight, fright and flight) induced by the intervention. However, for now, no significant effect on the sensorimotor network following BEATVIC was found. No differences in brain response between the BEATVIC group and the Befriending group following the intervention period were observed with GLM. This could be due to differences between both analysis methods: ICA is more sensitive in detecting task-related changes in fMRI signal than GLM because ICA uses a data-driven approach and can reduce noise in the final solution by separating artifacts from real fMRI signal [41]. With regard to behavioral data of the EF and the WoF task, formal RM ANOVAs also revealed no significant differences in RT and Acc between groups over time. However, post hoc we found a significant higher accuracy for the angry face condition in the BEATVIC group following the intervention compared to pre-treatment, and at post-treatment BEATVIC had significant higher accuracies for unambiguous emotion trials compared to the Befriending group.

VBM results revealed no differences between BEATVIC and Befriending, which is in line with a review by our group in which we found that an average weekly exercise frequency of at least two times a week might be the minimum to detect neural changes of physical activity interventions in psychosis [42, 43]. The current study suggests that an intervention consisting of physical activity one time (an hour) a week for a total of 20 weeks may be insufficient to evoke structural brain changes in psychosis.

Some limitations of this study should be mentioned. First, the relatively small sample size may have prevented current trend effects of ICA-based analyses and behavioral analyses to reach statistical significance. The lack of results of GLM-based analyses might also be partly explained by the sample size. Most previous fMRI studies on social cognition training or brain stimulation interventions in psychosis included around 20 participants [13, 16, 44, 45], which is still modest. Furthermore, treatment groups were heterogeneous with regard to age and illness duration. Although we chose to include a rather broad patient group to increase generalizability, future studies with larger sample sizes may investigate these potential confounding factors. Another limitation concerns the component selection. While the selected components included, respectively, visual regions, frontal regions and the insula as ROIs, none of the components included the amygdala. We selected the salience network based on an ROI mask including both the insula and amygdala. However, no other component included the amygdala. The salience network, left fronto-parietal network and sensorimotor network selected for the EF task showed a relative low correlation with the task compared to for example the visual network. However, previous published studies found similar correlations with a task [45, 46] or selected components based on spatial sorting only [47]. Furthermore, low correlations with a task might also be due to the nature of the particular task, for example, the brief presentation of stimuli. On a different note, BEATVIC targets several factors: face processing, and social cognition in general, but also self-esteem, illness insight and aggression regulation. These different elements complicate comparison with other interventions, for example those that focus on face processing only, and hinder inferences about which element is responsible for a certain effect. However, this is inevitable in clinical practice as most interventions consist of various aspects, as opposed to experimental laboratory studies in which one factor can be systematically manipulated and investigated at a time.

In summary, this study demonstrated that a body-oriented resilience therapy, aimed at preventing victimization by targeting associated factors such as difficulties with face processing, leads to increased activation of the salience network in response to threatening faces. The functional significance of this finding remains to be further established. Patients might have become more alert to threatening or potential dangerous faces following the intervention. This interpretation is supported by behavioral descriptives of the WoF that show reduced reaction times and more accurate responses for BEATVIC compared to Befriending over time. Statistically insignificant findings of the visual network and the sensorimotor network may be regarded as tentative and strongly warrant further investigation to allow for more definite conclusions. In short, our study shows that neuroimaging before and after a psychosocial intervention holds promise to generate hypotheses about the underlying mechanisms.

## Electronic supplementary material

Below is the link to the electronic supplementary material.

Supplementary file1 (DOC 50 kb)

Supplementary file2 (DOCX 253 kb)

Supplementary file3 (DOCX 17 kb)

Supplementary file4 (DOCX 17 kb)
