# Supplementary material for: Neural changes following a body-oriented resilience therapy with elements of kickboxing for individuals with a psychotic disorder: a randomized controlled trial
Source: Eur Arch Psychiatry Clin Neurosci. 2020 Jan 24;271(2):355–66. doi: 10.1007/s00406-020-01097-z (PMC7960594; doi:10.1007/s00406-020-01097-z)
Supplement: Supplementary file 1 — Supplementary file1 (DOC 50 kb) [file 406_2020_1097_MOESM1_ESM.doc]

**
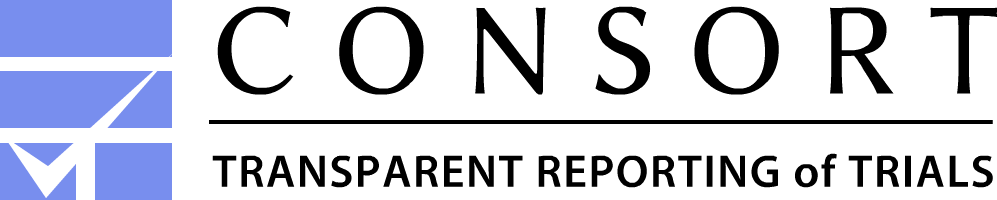
**

**CONSORT 2010 Flow Diagram**

**Allocation**

**Analysis**

**Follow-Up**

Analysed (n=14)
♦ Excluded from analysis (n=2; head movement > 3mm, n=1; technical problems)

Lost to follow-up (n=1; fear for MRI scanner, n=3; increased symptoms during assessment period )

Discontinued intervention (n=1; not motivated for intervention)

Allocated to BEATVIC (n=22)

♦ Received allocated intervention (n=22)

♦ Did not receive allocated intervention (n=0)

Lost to follow-up (n=1; fear for MRI scanner, n=1 increased symptoms during assessment period)

Discontinued intervention (n=2; not motivated for intervention)

Allocated to Befriending (n=19)

♦ Received allocated intervention (n=18)

♦ Did not receive allocated intervention (n=1, moved after randomization)

Analysed (n=13)
♦ Excluded from analysis (n=1; technical problems)

Randomized (n=41)
