# Supplementary figures and images for: Neural changes following a body-oriented resilience therapy with elements of kickboxing for individuals with a psychotic disorder: a randomized controlled trial
Source: Eur Arch Psychiatry Clin Neurosci. 2020 Jan 24;271(2):355–66. doi: 10.1007/s00406-020-01097-z (PMC7960594; doi:10.1007/s00406-020-01097-z)

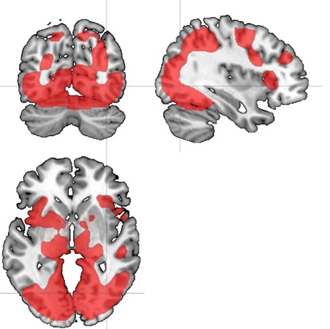

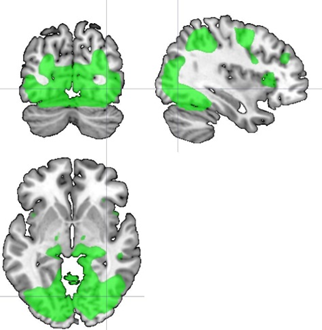


**Figure S3.** Task activation for angry>baseline (red) and fear>baseline (green).

Supplement: Supplementary file 2 — Supplementary file2 (DOCX 253 kb) [file 406_2020_1097_MOESM2_ESM.docx]
