# Supplementary material for: Neural changes following a body-oriented resilience therapy with elements of kickboxing for individuals with a psychotic disorder: a randomized controlled trial
Source: Eur Arch Psychiatry Clin Neurosci. 2020 Jan 24;271(2):355–66. doi: 10.1007/s00406-020-01097-z (PMC7960594; doi:10.1007/s00406-020-01097-z)
Supplement: Supplementary file 3 — Supplementary file3 (DOCX 17 kb) [file 406_2020_1097_MOESM3_ESM.docx]

Supplement 4. Detailed description of components.

EF

Component A (visual network, r=0,41) revealed a pattern of inferior, middle and superior occipital regions, the cuneus, the calcarine gyrus and the lingual gyrus. Component B (salience network, r=0,07) included the bilateral insula and anterior cingulate cortex. Component C (left frontoparietal network, r=0,07) consisted of the inferior, middle and superior frontal gyrus, the inferior parietal gyrus and the angular gyrus. Component D (sensorimotor network, r= 0,07) comprised the supplementary motor area, the precentral and postcentral gyrus, the paracentral lobule and part of the middle cingulate cortex.

WoF

Component A (medial visual network, r=0.62) consisted of the middle, inferior and superior occipital gyrus, lingual gyrus, calcarine gyrus and fusiform gyrus. Component B (salience network, r=0,21) comprised the bilateral insula and anterior and middle cingulate cortex. Component C (right fronto-parietal network, r=0,21) consisted of primarily left superior, middle and inferior frontal regions, as well as inferior and superior parietal gyri, angular gyrus and precuneus. Component D (sensorimotor network, r=0,14) comprised the supplementary motor cortex, the precentral gyrus, the postcentral gyrus, the paracentral lobule and the middle cingulate cortex.
