# Supplementary material for: Neural changes following a body-oriented resilience therapy with elements of kickboxing for individuals with a psychotic disorder: a randomized controlled trial
Source: Eur Arch Psychiatry Clin Neurosci. 2020 Jan 24;271(2):355–66. doi: 10.1007/s00406-020-01097-z (PMC7960594; doi:10.1007/s00406-020-01097-z)
Supplement: Supplementary file 4 — Supplementary file4 (DOCX 17 kb) [file 406_2020_1097_MOESM4_ESM.docx]

**Table S2. Brain areas activated in the emotional faces task.**

| **Contrast** | **Regions** | **Cluster size** | **T value** | **Z value** | **MNI coordinates** | | |
| --- | --- | --- | --- | --- | --- | --- | --- |
|  |  |  |  |  | **x** | **y** | **z** |
| Angry>baseline | Inferior/middle/superior occipital gyrus, inferior/middle frontal gyrus, inferior/superior parietal lobule, inferior/middle/superior temporal gyrus, lingual gyrus, (pre)cuneus, pre/postcentral gyrus, posterior/mid cingulate gyrus, fusiform gyrus, calcarine gyrus, culmen, insula, supplementary motor area, thalamus, amygdala, hippocampus | 12796 | 11.19 | 6.69 | 30 | -79 | -19 |
| Fear>baseline | Inferior/middle/superior occipital gyrus, inferior/iddle frontal gyrus, inferior/superior parietal lobule, inferior/middle temporal gyrus, lingual gyrus, (pre)cuneus, pre/postcentral gyrus, posterior/mid cingulate gyrus, fusiform gyrus, calcarine, insula, supplementary motor area, thalamus, hippocampus | 9496 | 11.29 | 6.74 | -6 | -88 | -4 |
|  | Superior/middle temporal gyrus, insula | 109 | 5.54 | 4.46 | 54 | -40 | 17 |
|  | | | | | | | |
